# The Impact of Cystic Fibrosis Algorithm Changes: A Case Study of Challenges and Strategies

**DOI:** 10.3390/ijns11030082

**Published:** 2025-09-19

**Authors:** Jerusalem Alleyne, Kenneth Coursey, Kimberly Noble Piper, Cynthia Cass, Michael Pentella

**Affiliations:** 1 State Hygienic Laboratory, University of Iowa, Coralville, IA 52241, USA; kenneth-coursey@uiowa.edu (K.C.); cynthia-cass@uiowa.edu (C.C.); michael-pentella@uiowa.edu (M.P.); 2 Iowa Department of Health and Human Services, Des Moines, IA 50319, USA; kimberly.noblepiper@hhs.iowa.gov

**Keywords:** newborn screening, cystic fibrosis, *CFTR*, newborn, neonatal screening, emergency preparedness

## Abstract

The State Hygienic Lab at the University of Iowa (SHL) performs newborn blood spot screening (NBS) for IA, AK, ND, and SD. In October 2022, we halted in-house *CFTR* DNA testing due to the unexpected nonperformance of our newly expanded variant panel. Samples were sent to a reference laboratory to ensure uninterrupted testing and by December 2022, SHL had selected an alternative test that enabled *CFTR* panel expansion as envisioned. However, due to circumstances beyond our control, test implementation was severely delayed, and in-house testing was paused. These events were consequential. Firstly, our prolonged utilization of reference labs and fees was a financial strain on the lab. Secondly, our timeliness decreased significantly, and lastly, these issues were burdensome for staff. The lab overcame these problems using three strategies: effective communication; technical expertise; and staff perseverance. Finally, in Aug 2023, SHL successfully resumed in-house testing. As state labs ponder major *CFTR* algorithm changes, such as the addition of next generation sequencing, the strategies we utilized can be useful during sudden setbacks. Our experience of replacing our *CFTR* assay underscores the importance of emergency preparedness and partnership within the NBS community.

## 1. Introduction

Newborn Screening (NBS) is a public health initiative that identifies newborns at risk for inherited or congenital disorders, facilitating early interventions that can minimize the impact of these disorders [1,2]. Every year, approximately four million newborns are screened in the US [2]. NBS is performed in all 50 US states via near-patient tests and dried blood spot (DBS) testing. NBS labs perform DBS testing. The guidance on which disorders labs should screen for comes from the Recommended Uniform Screening Panel (RUSP), which is produced by the US Department of health and human services (HHS) [1,2,3].

Cystic Fibrosis (CF) is one of the most common and debilitating inherited conditions in the US (~1 in 4000 US births) [4,5,6,7]. CF is an autosomal recessive multi-organ disease, caused by mutations in the Cystic Fibrosis Transmembrane Conductance Regulator (*CFTR*) gene [4,5]. The resulting dysfunction in *CFTR* protein leads to altered chloride transport across cell membranes. This causes less water to be attracted to the cell surface, causing the production of thick mucus in organs [5]. In the lungs, this mucus blocks the airways, allowing bacteria to become trapped and increasing susceptibility to infections. Mucus buildup also causes pancreatic insufficiency, a condition where the pancreas’ ability to release digestive enzymes is attenuated. This results in diminished nutrient absorption, so babies fail to thrive. Some males with CF also suffer from infertility [4,5]. Early treatment is critical to improve the quality of life and outcomes for CF patients.

The Iowa Newborn Screening Program (INSP) directs NBS for newborns in Iowa and comprises three entities: (1) Iowa Health and Human Services (Iowa HHS) [8], (2) The State Hygienic Lab at the University of Iowa (SHL) [9], (3) the University of Iowa Health Care Stead Family Department of Pediatrics (UIDP) [10]. Iowa HHS has a memorandum of understanding with SHL and UIDP. Together, these entities execute the goals of the INSP.

SHL has stood at the front line of public health matters in Iowa (IA) since 1904. Blood spot screening is performed exclusively at SHL’s facility in Ankeny, IA. The INSP’s follow-up team, medical consultants and genetic counselors (GCs) are associated with the UIDP, which is in Iowa City, IA. SHL runs a 365-day, two-shift operation, and conducts testing on weekends, holidays and at night, making it the only state public health lab with a dedicated night shift. In addition to IA, SHL conducts NBS testing for the states of Alaska (AK), North Dakota (ND) and South Dakota (SD) and screens ~70,000 newborns annually. AK, IA, ND and SD are all “one-screen” states, meaning newborns are screened once, 24–48 h after birth, unlike two-screen states that do a second screen 1–2 weeks after birth.

The INSP utilizes a three tier testing algorithm to screen for cystic fibrosis: (1) Immunoreactive trypsinogen (IRT) [5,6,7] with a fixed cutoff of ≥58 ng/mL (SHL is actively evaluating the benefit of a floating cutoff). (2) Samples over this cutoff are reflexed to *CFTR* deoxyribonucleic acid (DNA) screening [5,6,7]. Every month, ~130 samples are reflexed to *CFTR* DNA analysis and those with one or more variants are tested a second time. The results from the first and second run must match before they are released. (3) A sample will be reflexed to Sanger sequencing if the two second-tier runs produce discordant results, and this is performed by the Wisconsin State Laboratory of Hygiene.

In 2022, SHL staff met with CF clinicians/consultants, state coordinators, GCs and follow-up staff from IA, AK, ND and SD to discuss *CFTR* panel expansion. At that time, our “old test” was a lab-developed test (LDT). It was a qualitative genotyping assay, utilizing microfluidic cards. Based on a data-driven review performed by SHL, all entities agreed on the need to expand our 25-variant “limited” panel to effectively identify more variants in the four states. Once selected, a custom 42-variant “expanded” panel was ordered in July 2022. Following this event, we experienced two distinct phases of problems that prevented in-house testing for 9.5 months. Phase 1: the 42-variant “expanded” panel, sourced from vendor #1, was defective and could not be used for patient testing. This caused us to halt in-house testing and send our samples to a reference lab while we sought a replacement test. Phase 2: SHL selected a new test from vendor #2 but experienced major implementation delays beyond the lab’s control, due to administrative, logistical and technical issues. This necessitated the use of reference labs for longer than anticipated and delayed the resumption of in-house testing. Both problems/phases are described below.

Phase 1: When we received the 42-variant “expanded” panel (old test), problems were observed immediately. First, we discovered physical defects in many cards that prevented the entry of reagent and sample into some wells, resulting in no amplification in the affected wells. Next, there were significant test performance issues and inter-lot variability between the “limited” and “expanded” panels (old test), and we observed incorrect calls on the “expanded” panel (when compared to the “limited” panel). Workarounds for these problems became too numerous and unsustainable, so the “expanded” panel could not be validated according to SHL standards. Vendor #1 could not rectify the issue, and with the exhaustion of the “limited” panel looming, SHL suddenly and unexpectedly lost our ability to perform in-house *CFTR* DNA analysis. This prompted us to send all samples reflexed to second-tier testing to a reference laboratory, and we decided to source a new assay.

Phase 2: Once a new test from vendor #2 was selected, its implementation faced unexpected setbacks related to administrative (quotes and contracts), logistical (shipping of instruments) and technical delays. These setbacks stalled the resumption of in-house testing, requiring continued use of reference labs. Three consecutive reference labs were used to process ~1400 samples during the 9.5-month period based on their ability to accommodate our sample load. The fallout from phase 1 and 2 problems affected many functional aspects of the INSP, such as our timeliness which was decreased significantly, additional fees for reference labs, shipping fees, delayed reports, additional hours for staff and staff frustration and stress. Below, we describe the strategies taken to resume in-house *CFTR* DNA testing while also ensuring that *CFTR* screening for AK, IA, ND and SD babies was not interrupted.

## 2. Methods

### 2.1. Solving Phase 1 Problems

#### 2.1.1. Deciding to Change Our CFTR DNA Test

This extensive process began by immediately contacting vendor #1 when issues with the old 42-variant “expanded” panel were discovered. Numerous troubleshooting exercises were performed by the lab and vendor #1. Unfortunately, vendor #1 could not offer a solution, so there was a risk that the problems could reoccur. Our “limited” panel was nearly exhausted, so we would have been without an operational *CFTR* DNA assay in a matter of weeks. SHL determined that using the test in patient testing posed too high a risk and made the decision to: (1) shut down in-house testing after the 25–variant “limited” panel was exhausted, (2) send samples to a reference laboratory, (3) replace our *CFTR* DNA test.

#### 2.1.2. Reference Labs

To guarantee the seamless continuation of testing, reference lab testing was organized prior to the exhaustion of the “limited” panel. During the two phases of problems/problem solving (9.5 months), SHL utilized 3 reference labs consecutively as their capacity allowed (Minnesota: October–November 2022, Missouri: November–February 2023 and Indiana: February–August 2023). Before we switched to a new reference lab, the following tasks were coordinated:

- Enactment of existing memoranda of understanding (MOUs)/contracts and the establishment of new agreements. This involved the legal teams of both entities to determine the contract’s length (e.g., 1 year), renewal date and the duration of testing.
- Agreement on the price of testing per sample and whether repeat testing (to confirm detected variants) was an additional charge.
- Obtained information on the reference lab’s assay and variant panel. After the first reference lab, we ensured that subsequent labs used the same test/panel to minimize the impact on our patient reports, database and standard operating procedures (SOPs).
- Decided on the sample type to be sent (whole blood spots vs. punches).
- Ascertained the reference lab’s testing schedule, agreed on a preferred carrier and created a compatible shipping schedule (amended during holidays).
- Organized the secure delivery of reports and the return of residual blood spots to SHL.
- Labs exchanged the email addresses of staff involved in testing to ensure redundancy in the receipt of samples, results and correspondence.

#### 2.1.3. INSP Preparations

- It should be noted that only in-house *CFTR* DNA testing was halted at SHL, and no other tests were affected. Also, downstream NBS processes remained fully functional and unaltered (follow-up, GC and medical consultations, confirmatory testing, diagnostic and therapeutic approaches, etc.).
- SHL updated its laboratory information management system (LIMS) to reflect the variants on the reference lab’s panel (performed only once because the 3 reference labs ran the same test/panel).
- We amended patient reports to indicate that *CFTR* DNA analysis was performed at a reference lab (address and Clinical Laboratory Improvement Amendments [CLIA] number were included).
- Shipping SOPs were revised, and shipping fees were calculated.
- SHL and follow-up SOPs were updated to reflect the new variants, and staff were trained accordingly.

#### 2.1.4. Notifying Clients About Reference Labs (See Table 1)

- The initial communication was sent in October 2022, to the state coordinators of AK, IA, ND and SD, seeking approval for their samples to be tested by a non-SHL lab, as required contractually.
- The second major communication was to birthing facilities in January 2023. Owing to the increased turnaround time (TAT) for *CFTR* DNA results, the timeliness of patient reporting was delayed, resulting in a significant increase in call volume. To address this, SHL crafted a letter explaining the delays, that accompanied reports.
- The last major communication was in Aug 2023 to inform clients of the resumption of SHL’s in-house *CFTR* testing.
- In addition to these communiqués, *CFTR* assay challenges were routinesly discussed among the four states at our monthly ‘Quad state’ meeting.

### 2.2. Solving Phase 2 Problems: Implementing the New Test

#### 2.2.1. SHL Identified Three Main Attributes Required for the New CFTR DNA Test

Robustness, reliability and a good track record in the NBS community.

#### 2.2.2. Networking and Market Research

- This began in Oct 2022 at the Association of Public Health Laboratories (APHL) [11] NBS symposium and allowed SHL staff to meet with vendors and state labs with expertise in *CFTR* DNA analysis. This exercise yielded three viable options. After follow-up meetings with the respective vendors and examining the feedback from various state labs, only one vendor met our requirements, so a demonstration of the test at an experienced state lab was conducted in Nov 2022.
- In Dec 2022, SHL officially selected a 39-variant *CFTR* DNA test from vendor #2, with patented technology that utilizes PCR and flow cytometry.
- SHL relayed the urgency of test implementation to vendor #2. Both parties agreed on a feasible completion deadline (end of February 2023) and developed a plan of action for the following: (1) quotes and contracts, (2) instrument procurement and service contracts, (3) purchase of auxiliary equipment and consumables, (4) reconfiguration of lab space, (5) instrument installation, (6) test validation, (7) staff training. SHL also requested that these documents be given priority by University of Iowa departments (legal, accounting, procurement services) for expedited document reviews.

#### 2.2.3. Resolving the Issues Resulting from Delayed Test Implementation

- Despite our extensive planning, test implementation was not completed within the expected timeframe due to administrative (quotes and contracts), and logistical (instrument procurement/shipment) delays and technical issues.
- The administrative issues required a great deal of effort and communication with vendor #2 to be resolved. Once the equipment was installed, training of SHL staff by vendor #2 started. However, we experienced technical problems while trying to validate the test. Vendor #2 advised the lab on troubleshooting exercises which did not solve the problem. The lab then requested technical assistance from the U.S. Centers for Disease Control and Prevention (CDC), National Centers for Environmental Health, Newborn Screening and Molecular Biology Branch [12] and other public health labs.

## 3. Results

Various strategies were employed to mitigate the impact of the *CFTR* assay challenges.

### 3.1. Effective Communication with Clients and Vendors

**Figure 1:**
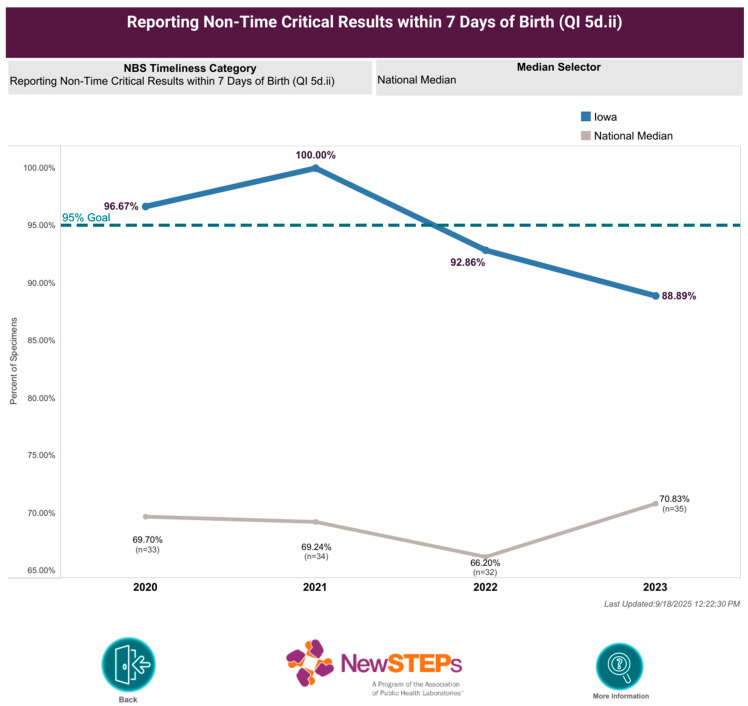
Decreased timeliness of non-time-critical reports caused by *CFTR* assay problems.

The most consequential outcome of in-house testing being paused was a significant decrease in our timeliness across all four states (for brevity, Iowa’s TAT metrics have been used as a representative dataset). This was reflected in Iowa’s report for non-time-critical results from the Newborn Screening Technical assistance and Evaluation Program (NewSTEPs) (Figure 1). The decline in timeliness can be attributed almost entirely to delayed *CFTR* results/reports. This necessitated continuous communication with our clients.

**Figure 2:**
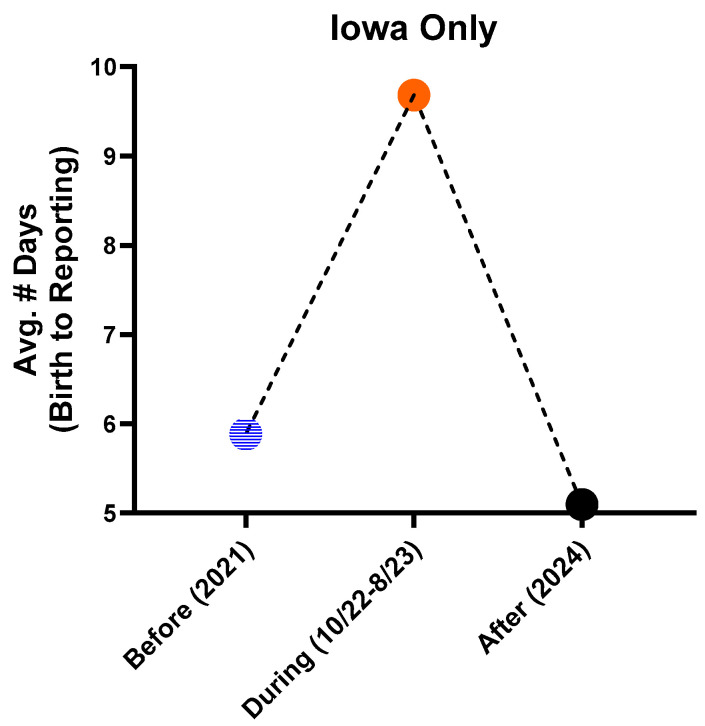
The average number of days for CF results (from birth to reporting) in Iowa, before in-house *CFTR* DNA testing was paused in 2021 (5.9 days), during the pause 10/22–8/23 (9.7 days) and after the new test was implemented in 2024 (5.1 days).

While a necessary process, shipping samples to reference labs became a limiting factor, because it influenced when samples could be tested by the reference lab according to its testing schedule. In many cases, thanks to a next-day delivery service, samples would be tested on the day of delivery or the following day. However, this could be affected by various factors such as delayed delivery, holidays/long weekends, instrument issues or the reference lab’s workload, etc. Sample logistics added ~2–4 days onto our TAT during the period of October 2022 to August 2023. Compared to 2021 (6 days), the average TAT for CF results in Iowa (birth to reporting) increased to 10 days (Figure 2).

Our decreased timeliness caused a significant uptick in call volume from clinics and community providers. The INSP has established performance metrics used to monitor the timeliness of results reporting. The additional time from birth to reporting was an outlier in our existing targets. The extended TAT did not go unnoticed by our clients who called to inquire about delayed reports and were informed that *CFTR* results were still pending. Between the follow-up team and the lab’s client services team, there were approximately 4–6 such enquiries per week. The lab’s letter to birthing facilities (Table 1), calls and emails with clients alleviated the situation. However, a return to normal call volumes was only achieved when in-house testing resumed, which improved our TAT for CF results to an average of 5 days in 2024 (Figure 2).

Another effective communication medium was our monthly “Quad state” meeting that is held virtually to discuss topics that affect the four states. Attendees included the AK, IA, ND and SD NBS state coordinators, SHL leadership, lab staff, information technology staff, follow up, the INSP Medical Director and other NBS personnel on an ad hoc basis. This medium allowed SHL to provide answers and updates on the *CFTR* assay challenges to a broad range of clients all at once.

In addition to clients, SHL staff regularly liaised with vendors to report testing issues, seek updates and work towards swift resolutions. During the troubleshooting of the problems with the old test and during delays in the new test’s implementation, SHL staff reiterated the matter’s urgency and that it was causing the lab to incur additional costs for reference lab fees and shipping. This ensured that these issues remained a primary focus for the vendors.

### 3.2. SHL Utilized In-House and External Technical Expertise to Resolve CFTR Assay Issues

While SHL performed the troubleshooting exercises recommended by both vendors #1 and #2, they did not resolve the problems we experienced. Therefore, the lab enacted two concurrent strategies. Firstly, we conducted our own controlled troubleshooting experiments. Secondly, we consulted external experts for technical assistance who provided an invaluable injection of fresh ideas, assay SOPs, quality control samples, assay experience and guidance. These two strategies were vital, especially during new test implementation, because we realized that our technical issues stemmed from discrepancies in the training instructions that we received from vendor #2.

Subsequently, we overcame the many hurdles and delays to successfully launch the new test, which met our needs (Table 2). Though there were other tests with larger variant panels on the market, there were three main factors that precluded us from using them: cost, reliability/reputation and a more complex workflow, which would require major facility changes and additional personnel. The new test is a 39-variant panel as compared to the 42-variants on the defunct “expanded” panel. However, CF clinicians, GCs and state coordinators were satisfied that the two panels were almost identical and the new panel included highly desired variants such as *M1101K* that is found in the Hutterites of SD [13]. Immediately after the launch of our new *CFTR* DNA assay, SHL received a Molecular Assessment Program (MAP) visit from the CDC, which provided invaluable quality assurance oversight.

### 3.3. Staff Initiative and Collaboration with the NBS Community Helped Resume In-House Testing

**Figure 3:**
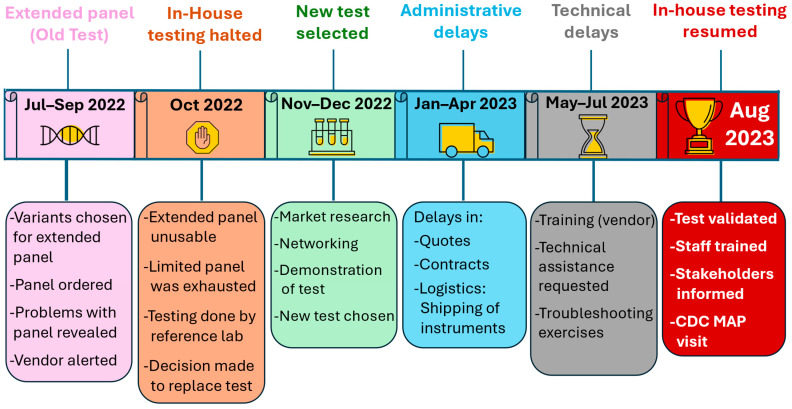
Timeline of events for SHL’s pause of in-house *CFTR* DNA testing.

INSP staff (SHL staff, follow-up staff, GCs and medical consultants) regularly went above and beyond when solving problems and providing excellent customer service. SHL staff, who bore the responsibility of test implementation, were committed, creative, persistent, resilient and calm when facing challenges. Thanks to these combined efforts, in August 2023, in-house testing resumed after 9.5 months (Figure 3).

## 4. Discussion

Two major events affected *CFTR* DNA testing at SHL from October 2022–August 2023: the nonperformance of our LDT and the prolonged implementation of its replacement. However, we also experienced triumphs, unforeseen benefits and learned many valuable lessons.

Our greatest success was preventing mass panic across four states. During any tumultuous period, the tone and contents of correspondence are vital factors in relaying a message adeptly while maintaining calm. SHL’s correspondence with clients was clear, concise, honest and informative, while retaining an appreciative and optimistic tone (Table 1). Conversely, when pursuing resolutions to problems, the lab’s correspondence with vendors was cordial and respectful, but was also persistent and always highlighted the impact of delays on our TAT and the fees associated with the protracted use of reference labs.

The feedback received from clients was positive, making this approach successful. Clients reported that they were appreciative of regular updates, prompt responses to questions, SHL’s decisive decision making and supplemental progress reports. Another vital factor was the relationship between SHL and its ‘Quad State’ partners. This special and close-knit alliance often resembles that of a family more than that of contractor and contractee. The impact of the compassion, understanding and empathy extended to SHL staff was profound and of immeasurable value.

The new test’s delayed implementation also provided a silver lining. By utilizing reference labs running the same test we later adopted, SHL was afforded a bird’s eye view of the test’s capabilities, data analysis procedures and other features. Moreover, SOPs from state labs gave us insight into how different labs resolved test-specific issues. This gave us an advantage when formulating our own SOPs and test workflows. For instance, we observed a rare testing event at two reference labs that caused discordant results, so we introduced Sanger sequencing as a third-tier test (performed by the Wisconsin State Laboratory of Hygiene) to prevent potential delays to our TAT, should it occur at the SHL.

Another strength was our ability to trust our own expertise and seek external assistance/oversight. In particular, the CDC [12] was an intermediary during SHL’s outreach with reference labs, and together they provided invaluable technical knowledge during our technical troubleshooting efforts (old and new test). Furthermore, days after launching the new test, the CDC provided further technical oversight of both the new test and SHL’s NBS Molecular section via a MAP visit. This exercise highlighted continuous quality improvement opportunities and reassured the lab and its stakeholders of our good test performances and the quality of results. The role of partnership and collaboration within various NBS entities is a theme of this work that cannot be overstated. The NBS community is very collaborative, and because of this, SHL did not hesitate to seek assistance.

Nevertheless, despite our best efforts, there were unavoidable issues that we could not resolve, such as the slower TAT while in-house testing was paused, and the expense of reference lab fees. The failure of vendor #1’s assay/microfluidic cards was unexpected, but such an occurrence is not unprecedented. In 2016, 53.4% of NBS labs performing *CFTR* DNA testing used assays/microfluidic cards developed by Hologic [14]. However, physical defects with the microfluidic cards led to them being recalled suddenly and the tests were discontinued [15,16]. Some labs switched to other assays [16], while others (like SHL), were forced to send samples to reference labs [17].

With the benefit of hindsight, SHL examined many of its processes, identified areas for improvement and learned many beneficial lessons. Firstly, our established MOU with the MN state lab allowed prompt coordination of testing when we could no longer run our old test. Therefore, SHL has built upon this by maintaining the MOUs with the other reference labs. Secondly, SHL’s emergency preparedness policies and procedures are under review. While our second-tier *CFTR* DNA test was paused, first-tier IRT testing remained unaffected, so it was not a triggering event for the INSP’s Continuity of Operations Plan (COOP) [18]. Also, this scenario was not specifically outlined in SHL’s Emergency Management Assistance Compact (EMAC) plan.

Subsequently, unlike other SHL partners, birthing facilities were not immediately alerted in October 2022 when in-house *CFTR* DNA testing was paused. To address this oversight, SHL has expanded its COOP team to include personnel involved in resolving *CFTR* testing issues, to gain fresh perspectives and identify additional blind spots in existing policies. Lastly, the importance of good vendor relationships to emergency preparedness was highlighted, given our experiences with vendors #1 and #2. SHL has revamped its strategies for handling vendor-related issues, so that they are swiftly escalated to SHL administration, who can intervene faster to advocate for the lab and staff. This improved approach has been used to successfully resolve subsequent vendor-related problems.

The NBS landscape is on the cusp of major metamorphosis. Advancements in therapies and testing methodologies have resulted in the addition of more tests to the RUSP [3], now more than at any time previously. Also, the utility of next generation sequencing (NGS) in NBS is already being examined [19,20] and encouraged by organizations like the CF Foundation [21,22]. NGS has the potential to revolutionize NBS. However, its incorporation into any NBS algorithm will be a major undertaking. Our experience, the strategies employed and lessons learned, especially those related to contingency planning and fostering connections within the NBS community, can undoubtedly be helpful to any lab embarking on major algorithm changes like NGS. In conclusion, we have described how best laid plans can be derailed by unexpected obstacles and provided a useful roadmap on how to prepare for and navigate rough waters amidst an unexpected ‘storm’.

## Figures and Tables

**Table 1:** Core contents of SHL correspondence to clients.

Contents of Communication	Recipient(s)
A concise and straightforward explanation of the problem and that pausing in-house testing was a necessary but temporary measure to ensure reliable test results	All Clients
Reassurance that SHL would implement a stable, robust and reliable alternative	All Clients
A request by SHL for approval to send samples to reference lab(s)	AK, IA, ND and SD state partners
Information on the use of a reference lab to perform *CFTR* DNA testing	All Clients
A list of variants on the reference lab’s panel	AK, IA, ND and SD state partners
An explanation of the decreased timeliness of patient reports awaiting *CFTR* DNA results	Birthing Facilities
Assurance that critical result reporting remained unaffected	Birthing Facilities
Explanation of SHL’s ongoing efforts to resolve issues	All Clients
Resumption of in-house testing and SHL’s appreciation of clients’ patience and understanding	AK, IA, ND and SD state partners

**Table 2:** A comparison of our two *CFTR* DNA assays (old vs. new).

Test Characteristics	Old Test (Custom Microfluidic Plates)	New Test (39-Variant Panel)
# Variants	Limited 25-variant panel (the defunct 42-variant expanded panel failed validation and was never used for patient testing)	Provided suitable detection of CF-causing variants in all 4 states
Vendor and Technology	Vendor #1—Single nucleotide polymorphism genotyping test	Vendor #2—Patented technology utilizing PCR and flow cytometry
Customization	Custom panel allowed greater flexibility in choosing variant panel	Variant panel is set by manufacturer
Physical footprint of test equipment/workspace	Workspace and instrument spaces were shared with other molecular tests	Needed larger footprint for test workflow, requiring lab reconfiguration
Cost of test	Relatively inexpensive	Almost double the cost of old test
FDA approved test	No (LDT)	Yes
Test runtime	~4 h	Longer test (~6 h)
Reliability and Robustness of assay	Physical anomalies on assay plates and lot-to-lot performance variability observed	Robust, reliable and used by many US NBS state labs
Test type: qualitative/quantitative	Qualitative results, some manual formatting of data analysis software necessary	Qualitative results, data analysis software formatted by the manufacturer
Automation/ease of use	Mostly automated	Less automated: numerous manual pipetting and mixing steps
Training	Training is relatively simple	Training can be prolonged (depending on technical skill)

## Data Availability

The original contributions presented in this study are included in the article. Further inquiries can be directed to the corresponding author Jerusalem Alleyne at jerusalem-alleyne@uiowa.edu.
